# Prognostic significance of the preoperative neutrophil-to-lymphocyte ratio patients with giant cell tumor of bone

**DOI:** 10.4314/ahs.v21i3.35

**Published:** 2021-09

**Authors:** Aliekber Yapar, İsmail Burak Atalay, Mehmet Ali Tokgöz, Coşkun Ulucaköy, Bedii Şafak Güngör

**Affiliations:** 1 Department of Orthopaedics and Traumatology, Dr. Abdurrahman Yurtaslan Ankara Oncology Training and Research Hospital, Ankara, Turkey; 2 Department of Orthopaedics and Traumatology, Dr. Nafiz Korez Sincan State Hospital, Ankara, Turkey

**Keywords:** Giant cell tumor of bone, neutrophil-to-lymphocyte ratio, prognostic significance

## Abstract

**Objective:**

To evaluate the prognostic significance of neutrophil-to-lymphocyte ratio (NLR) in giant cell tumor of bone (GCT).

**Methods:**

The patients with GCT were identified in the hospital records and pre-treatment complete blood count results were acquired retrospectively. Whether preoperative NLR lymphocyte-to-monocyte ratio (LMR) and platelet-to-lymphocyte ratio (PLR) values had prognostic significance in predicting recurrence was evaluated by Receiver operating curve (ROC) analysis. Furthermore, the prognostic value of NLR was evaluated by Multivariable Cox Regression analysis.

**Results:**

There were 96 patients with GCT. It was found that only NLR values had prognostic significance for predicting recurrence (AUC:0.647; 95% CI:0.533–0.762; P=0.021). The statistically significant cut-off value of NLR for predicting recurrence was ≥2.25. NLR was ≥2.25 in 51% (n = 49) of patients. Multivariable analysis showed that NLR ≥2.25 (HR=2.9, 95% CI:1.3–6.6; p=0.009) and lung metastasis (HR=7.9, 95% CI:2.2–28.2; p=0.001) were independent factors of recurrence. In patients with lung metastasis and patients with NLR ≥2.25, recurrence was observed in a sooner period (Log rank test; p=0.001; p=0.009, respectively).

**Conclusion:**

Our findings showed that NLR is a new and promising inflammation-based prognostic factor in GCT patients.

## Introduction

Giant cell tumor of bone (GCT) is a primary bone tumor that accounts for about 15% of benign tumors and 3% to 8% of all bone tumors^1^. GCT is a locally aggressive and benign bone tumor that can cause progressive bone damage and loss of function in the joints. Although curettage is an acceptable treatment modality with the additional use of local adjuvant therapies, wide resection is still an option for better regional surgical control of the disease. Lung metastasis (5% of cases) has been reported in the literature, but it is rare for GCT to conclude a life-threatening clinical outcome with systemic spreading^2^.

It is demonstrated that major factors for preventing local recurrence and systemic spread are surgical technique (more common after curettage) and usage of local adjuvant therapy (High-speed burring, cementing, phenol, hydrogen peroxide, nitrogen, and alcohol, etc.). Significant non-surgical prognostic factors for recurrence of GCT have been identified including age, tumor placement, Campanacci classification, tumor enlargement to soft tissue, and presence of pathological fracture^3^,^4^.

It is reported that some hematological parameters, like C reactive protein (CRP)^5^, platelet volume^6^, neutrophil to lymphocyte ratio (NLR)^7^, platelet to lymphocyte ratio (PLR)^8^, and lymphocyte to monocyte ratio (LMR)^9^ can be associated with outcomes of inflammatory, auto-immune and neoplastic diseases^10^. However, the value of NLR, LMR and PLR is not well understood in GCTs. In this study, the hypothesis was formed that these hematological parameters, associated with many malignant tumors, may be prognostic markers of local aggressive tumors such as GCT. To clarify this issue, we aimed to analyze the prognostic significance of preoperative NLR, LMR, and PLR dynamics in the differential diagnosis between recurrence and progression in GCTs.

## Materials and Methods

The study protocol was organized retrospectively on patient files and no additional interventional procedure was performed.Local committee approval was obtained (approval year and number are 2020/88). Clinical and demographic data of patients were reviewed from the hospital data system. Age, sex, tumor location, side, preoperative complete blood count results, date of diagnosis, last follow-up date, preferred surgical strategy, status of lung metastasis, presence of recurrence and recurrence date were reviewed from patient records. Patients who were diagnosed with GCT by histopathological methods, being performed no previous treatment that could change their blood values, having at least 12 months the follow-up period, and having available medical records were included to study. Patients with elevated C-reactive protein and procalcitonin results, those with diabetes mellitus, infections disease, rheumatologic diseases, and other inflammatory diseases, any blood disease, and those with missing medical records were excluded from the study. The total number of patients diagnosed with GCT but not included in the study was 18. NLR and PLR were calculated as the absolute count of neutrophils and platelets respectively, divided by the absolute lymphocyte count. LMR was calculated as the absolute count of lymphocyte divided by the absolute monocyte count.

### Statistical analyses

All statistical analyses were performed using Statistical Package for the Social Sciences (SPSS) version 22.0 software (Chicago, USA), and a p value <0.05 was considered to be statistically significant. In statistical analysis, categorical variables are given as numbers and percentages, and continuous variables are presented as mean ± standard deviation (SD) and as median (Interquartile Range: IQR) for descriptiveanalyses. Chi-squared tests were used to compare the categorical variables in independent groups. The conformity of continuous variables to normal distribution was evaluated using visual (histogram and probability graphs) and analytical methods (Kolmogorov-Smirnov / Shapiro-Wilk tests).

Normality analysis revealed that all data sets were not normally distributed. Mann-Whitney U test was used to compare the data sets that were not normally distributed for variables. Whether preoperative NLR, LMR, and PLR values had identified optimal cut-offs for recurrence was evaluated by Receiver operating curve (ROC) analysis. AUC (Area under curves) and cutoff values obtained from ROC analysis and sensitivity, specificity, positive predictive value (PPV), and negative predictive value (NPV) of these cutoff values were presented. Effect of some independent predictors of recurrence was evaluated by Multivariable Cox Regression analysis. In the Cox regression analyses, NLR was included as a categorical variable (high = NLR ≥ 2.25 and low <2.25 NLR) and log-transformed continuous variable (logNLR). Cox regression analysis results were presented with Hazard Ratio (HR) and 95% Confidence Interval (CI).

## Result

### Patient characteristics

There were 96 patients diagnosed as giant cell tumor of bone between January 2002 and December 2018. Forty-three of the patients were males and 53 were females with a median age of 28.5 years (IQR, 20.3 to 37.8). The median follow-up of patients included in the analysis was 61.3 (IQR, 37.3 to 81) months. Sixty-two of GCTs (64.6%) were localized in the lower limb, 28 (29.1%) in the upper limb and 6 (6.3%) in the pelvic girdle. The patients' baseline characteristics are summarized in Table 1.

**Table 1:** Baseline Demographics

Characteristic	Total N=96
**Age, year**	
Mean ± SD	30±12.5
Median (IQR)	28.5 (20.3–37.8)

**Sex, n(%)**	
Female	53 (55.2)
Male	43 (44.8)

**Side, n(%)**	
Right	49 (51)
Left	47 (49)

**Localization, n(%)**	
Pelvic ring	6 (6.3)
Femur distal	25 (26)
Femur proximal	6(6,3)
Tibia distal	3(3,1)
Tibia proximal	17 (17.7)
Fibula proximal	8 (8.3)
Humerus proximal	4 (4.2)
Radius distal	16 (16.7)
Ulna	3 (3.1)
Others	8 (8.3)

**Surgical Treatment, n(%)**	
Curettage +Bone grafting	65 (67.8)
Curettage +Bone grafting + Fixation	12 (12.5)
Curettage + Cementing	3 (3.1)
Curettage + Cementing + Fixation	3 (3.1)
Wide resection + Reconstruction with tumor prosthesis	8 (8.3)
Wide resection + Reconstruction with fibular grafting	5 (5.2)

**Follow-up time, months**	
Mean ± SD	61.3±33.1
Median (IQR)	56 (37.3–81)

**Lung Metastases**	
Yes	4 (4.2)
No	92 (95.8)

**Recurrence, n(%)**	
Yes	30 (31.2)
No	66 (68.8)

Patients were divided into two groups according to recurrence (Table 2). The median follow-up period in patients with recurrence was 68 months (IQR, 37.8 to 92.3), and median months were 53.5 (IQR, 35.5 to 71) for patients without recurrence (p = 0.069). In patients with recurrence, median recurrence time was 37 (IQR, 23 to 50.5) months. As recurrence surgery, wide resection and reconstruction with tumor prosthesis in 14 patients, curettage and bone grafting in 8 patients, curettage and internal fixatioin 2 patients, curettage and cementing and internal fixation in 1 patient, curettage and bone grafting and fixation in 3 patients, amputation in 1 patient, and wide resection and reconstruction with fibular grafting in 1 patient were performed. Pulmonary metastasis was found in 10% of patients with recurrence and 1.5% of patients without recurrence, this difference was not statistically significant (p = 0.089). Recurrence occurred in 29 of 83 (34.9%) patients who had curettage, whereas only 1 of 13 patients who had wide resection had recurrence. Hemoglobin, LMR, and PLR values were similar in both groups (p> 0.05). NLR values were found to be higher in patients with recurrence than those who did not develop (p = 0.021).

**Table 2:** Evaluation of Patients Groups

N=96	Recurrence		
		
	Yes (n=30)	No (n=66)	P
**Age, years**			0.279*
Median (IQR)	30.5 (22.5–39.3)	28 (19–36.3)	

**Sex, n(%)**			0.391**
Female	19 (63.3)	34 (51.5)	
Male	11 (36.7)	32 (48.5)	

**Side, n(%)**			0.720**
Right	14 (46.7)	35 (53)	
Left	16 (53.3)	31 (47)	

**Follow-up time, months**			0.069*
Median (IQR)	68 (37.8–92.3)	53.5 (35.5 - 71)	

**Surgical Treatment, n(%)**			0.058**
Curettage	29 (96.7)	54 (81.8)	
Wide resection	1 (3.3)	12 (18.2)	

**Lung metastasis, n(%)**			0.089**
Yes	3 (10)	1 (1.5)	
No	27 (90)	65 (98.5)	

**Hg, gram/dl**			0.171*
Median(IQR)	13.5 (12.1–14.5)	14.2 (12.5–15.2)	

**NLR**			**0.021** *
Median(IQR)	2.5 (2.1–3.1)	2 (1.6–2.7)	

**PLR**			0.293*
Median(IQR)	150.8 (110.4–193.2)	134.6 (108.6–183.5)	

**LMR**			0.387*
Median(IQR)	4.4 (3.6–5.7)	5 (3.5–6.4)	

NLR: Neutrophil-to-Lymphocyte Ratio; PLR: Platelet-to-Lymphocyte Ratio; LMR: Lymphocyte-to-Monocyte Ratio; Hg: Hemoglobin IQR: Interquartile Range

* Mann-Whitney U test

** Chi-Square Test

### ROC curve for to identify optimal cut-offs

**Figure 1:**
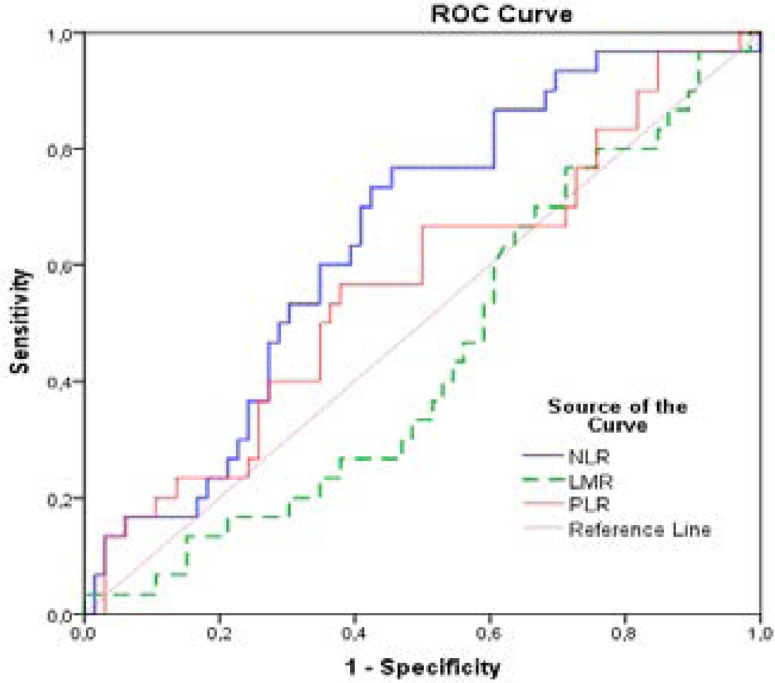
ROC-Curve for NLR, LMR and PLR as predictors of recurrence, n=96

ROC analysis was performed to determine whether NLR, LMR, and PLR values were statistically significant cut off values in predicting recurrence during follow-up(Figure 1). As a result of this evaluation, it was found that only NLR values had significance for recurrence (AUC: 0.647; 95% CI: 0.533–0.762; P = 0.021). The statistically significant cut - off value of NLR for predicting recurrence was ≥2.25. The results of ROC analysis of LMR, PLR, and NLR values were presented at Table 3.

**Table 3:** Statistical parameters of various preoperative NLR, LMR, and PLR values for predicting recurrence in patients

Parameter	AUC(95% CI)	P	Cut-off	Sensitivity	Specificity	PPV	NPV
NLR	**0.647 (0.533–0.762)**	**0.021**	**≥2.25**	70.0%	60%	42.9%	80.9%

LMR	0.445 (0.324–0.565)	0.387	≥4	63.3%	37.9%	31.7%	69.4%

PLR	0.567 (0.442–0.693)	0.293	≥133.5	66.7%	50%	36.5%	75%

PPV Positive Predictive Value, NPV Negative Predictive Value

NLR: Neutrophil-to-Lymphocyte Ratio; PLR: Platelet-to-Lymphocyte Ratio; LMR: Lymphocyte-to-Monocyte RatioAUC: Area Under Curve; CI:Confidence Interval

### Evaluation of Prognostic factors

Cox regression analysis was used to evaluate the prognostic value of the independent factors for recurrence. In the univariable analyses between two groups with and without recurrence, variables with p <0.1 were included in the multivariable cox regression analysis model. The univariable analysis showed that the factors of P <0.1 were found to be lung metastasis, surgical treatment, and NLR. The effect of parameters of recurrence was evaluated by Multivariable Cox Regression analysis by adjusting age and gender. It was determined that HR for lung metastasis was 7.9 (95% CI: 2.2–8.2; p = 0.001), HR for patients with NLR ≥2.25 was 2.9 (95%CI: 1.3–6.6; p=0.009) and HR for curettage was 5.2 (95% CI: 0.7–38.4; p = 0.109). Accordingly, patients with lung metastasis increased 7.9 fold risk of recurrence. In patients with NLR ≥2.25, the risk of recurrence increased 2.9 fold compared to patients with NLR <2.25. (Table 4, model 1). The Multivariable Cox Regression analysis model (model 2) was re-established by including LogNLR. As the categorical variable of NLR, LogNLR was also found to be associated with recurrence (HR = 2.3, 95% CI = 1.02- 5.3, P = 0.044) (Table 4, model 2).

**Table 4:** Multivariable COX regression analysis on risk factors for recurrence during the follow-up period in patients

	Univariable Cox regression		Multivariable cox regression analysis model-1*		Multivariable cox regression analysis model-2*	
	
	Crude HR (95% CI)	P	Adjusted HR (95% CI)	P	Adjusted HR (95% CI)	P
**Lung metastasis (ref: no)**	6.4 (1.9–21.6)	**0.003**	7.9 (2.2 – 28.2)	**0.001**	6.02 (1.7 – 20.8)	**0.001**

**NLR ≥ 2.25 (ref: < 2.25)**	2.7 (1.2 – 6.0)	**0.013**	2.9 (1.3 – 6.6)	**0.009**	2.3(1.02 – 5.3)**	**0.044**
**Surgical Treatment,** **Curettage(ref: Wide resection)**	6.3 (0.9–46.6)	0.07	5.2(0.7 – 38.4)	0.109	5.8(0.8 – 42.9)	0.87

HR: Hazard Ratio; CI:Confidence Interval; NLR: Neutrophil-to-Lymphocyte Ratio

* Cox regression analyses adjusted for sex and age

** NLR (per 1 log NLR higher)

**Figure 2:**
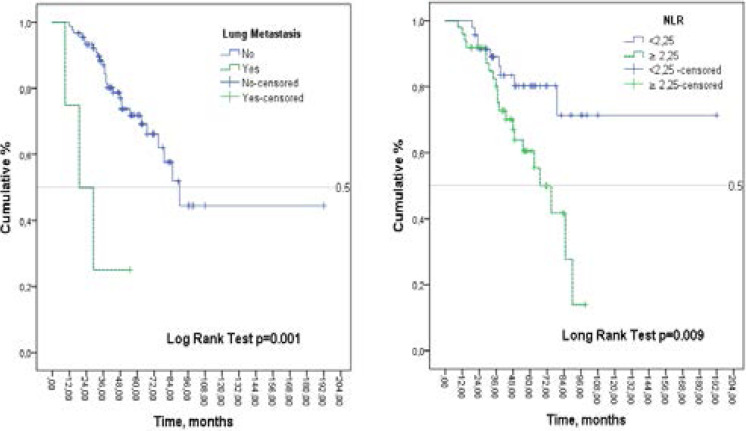
Kaplan-Meier curves for recurrence

Recurrence rates of the patients according to lung metastasis status and NLR were also evaluated by the Kaplan Meier method and Log-Rank test (Figure 2). In patients with lung metastasis and in patients with NLR ≥2.25, recurrence was observed in a shorter period (Log rank test; p = 0.001; p = 0.009, respectively).

## Discussion

Differences in parameters of complete blood count can reflect the dynamic balance between a tumor suppressor and oncogenic parameters. NLR, LMR, and PLR are simple derivatives of routine blood counts and have been identified as significant prognostic factors in the wide numbers of patients with inflammatory and neoplastic disorders^11^–^15^. The idea of using blood count parameters as a prognostic factor has gained increased interest in diversified musculoskeletal tumors^16^–^20^. However, this is one of the premise studies to investigate the relationship between preoperative hematological parameters and prognostic significance of GCT.

Local tumor control is difficult because of GCT's aggressive behavior. In some patients, functional statuses of joints and bones have to be sacrificed^21^,^22^. Recurrence rates of GCTs are highly controversial and major factors are about surgical technique. Recurrence rates of GCT range from 27 to 65% for isolated curettage, 12–27% for curettage with local adjuvant, and 0 - 12% for wide resection. Lung metastases with an often latent behavior occur in 2.1 – 6.6% of patients, mostly with complex or recurrent GCTs^23^.

In addition to that, Adjuvant therapies like denosumab and zoledronic acid for stabilization of local and metastatic GCT have been reported, although it is still controversial^23^. On the other hand, after the end of denosumab therapy, cases with malignant transformation were presented in the literature, especially after curettage^24^,^25^.

Intralesional curettage is a preferred technique for GCTs by several surgeons but it is challenged because of local recurrence risk. Residual tumor tissue is associated with local recurrence and is a vital concern when performing intra-lesional procedures. Non-surgical prognostic factors in bone lesions are investigated for predicting recurrence clinically, radiologically^26^,^27^, and pathologically^28^. However, finding an easy, non-invasive, and cost-effective prognostic factor for recurrence remains a major challenge for surgeons.

The NLR, LMR, and PLR can be determined simply from hematologic cell counts and obtained from results of blood tests that are routinely studied for many reasons in many medical institutions. It is demonstrated that NLR, LMR, and PLR are significantly associated with the outcome of many disorders. It is reported that preoperative NLR is an easy and cost-effective predictor for relapse in pigmented villonodular synovitis of the knee which is a tumor with a locally aggressive behavior like GCT, joint after arthroscopic surgery combined with local radiotherapy^20^.

GCT is a local destructive tumor of mesenchymal stromal cells; monocytic, mononuclear cells of myeloid lineage; and characteristic osteoclast-like, multinucleated giant cells^29^. It was observed that elevated levels of NLR were determined in patients with myeloproliferative neoplasms which were in the same stromal group of cells as GCT^30^,^31^. Besides, the prognostic role of NLR has been documented in multiple cancers from different stem cell lineage-based solid tumors like ovarian, breast, kidney, lung, colorectal^32^,^33^.

In the current study, we aimed to clarify the prognostic significance of NLR, LMR, and PLR in patients with GCT, like previous studies about those parameters in several disorders. It was found that NLR is statistically associated with the recurrence of GCT. In the ROC analysis, the AUC value of NLR was significantly higher in patients with recurrence, it was determined that NLR could be given as more accurate prognostic information about GCT. However, the prognostic value of LMR and PLR was not sufficient to predict the recurrence.

The possible limitations of this study include the retrospective, single-center nature of the study. Other limitations are that, first, blood cell counts are variables that could change rapidly and be affected by many factors, and only one pretreatment value was used in this study. Secondly, the long-term prognosis of GCT is influenced by many factors and the effect of these factors could not be determined due to the retrospective design. Lastly, there was heterogeneity in the treatment of these patients that could potentially cause bias.

The factors that make this study valuable are the relatively large sampling among studies on GCT patients and multivariable analyzes have shown that NLR remains an independent prognostic factor.

## Conclusion

Our findings showed that NLR was a promising inflammation-based prognostic factor in patients with GCT. Detection of NLR value above 2.25 before treatment was determined as a poor prognosis indicator. Another finding of the current study was that NLR could be a distinctive factor to be superior to that of other inflammatory markers, including LMR and PLR. Many different conditions determine the prognosis of neoplastic diseases, and NLR could be just one of them. NLR values cannot be a decision-maker alone in patients with GCT, but they should be one of the variables to be considered while making a treatment timing and strategy. Future multicenter prospective studies are needed to validate our findings and to investigate the value of the combined use of these inflammatory markers to prolong the remission period of patients with GCT.
